# Supplementary material for: Molecular architecture of the tumor microenvironment caused by BRCA1 and BRCA2 somatic mutations in human lung adenocarcinoma
Source: eLife. 2026 May 26;15:RP110662. doi: 10.7554/eLife.110662 (PMC13211877; doi:10.7554/eLife.110662)
Supplement: Figure 7—source data 1. [file elife-110662-fig7-data1.pdf]

Supplementary Figure. Uncropped immunoblot images

A. LDHA, S100A10, GAPDH shRNA (Corresponding to Figure 7D).

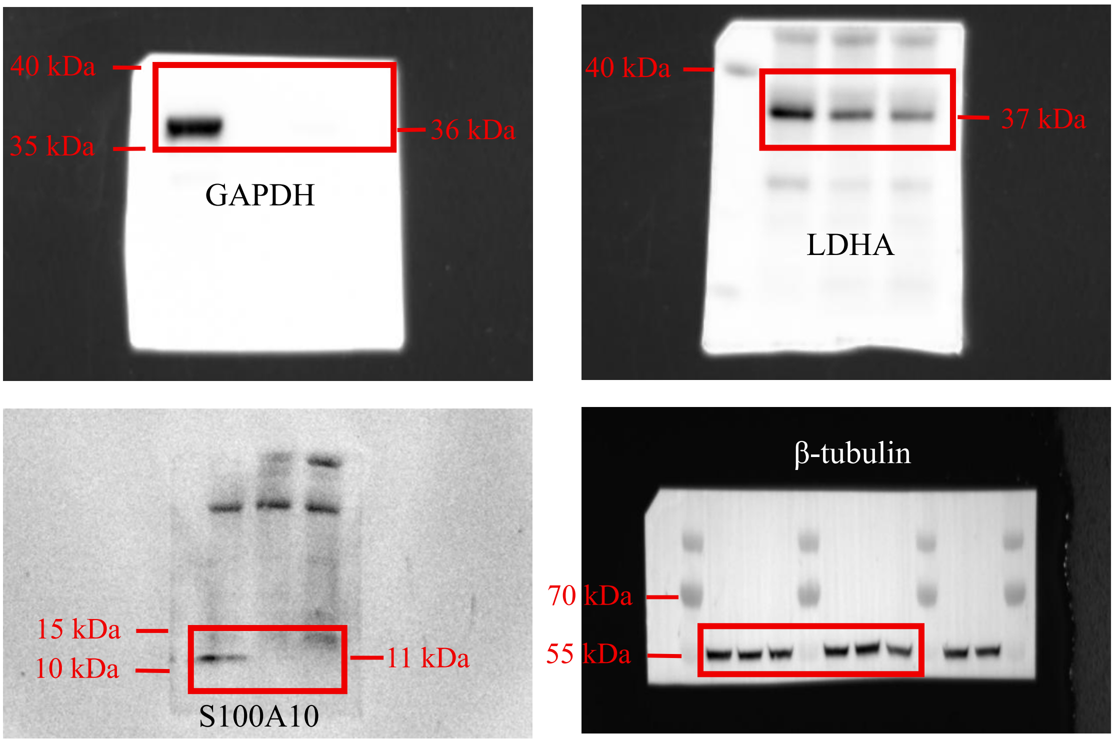

B. Vorinostat & Belinostat Drug treatment 48h (Corresponding to Figure 7I).

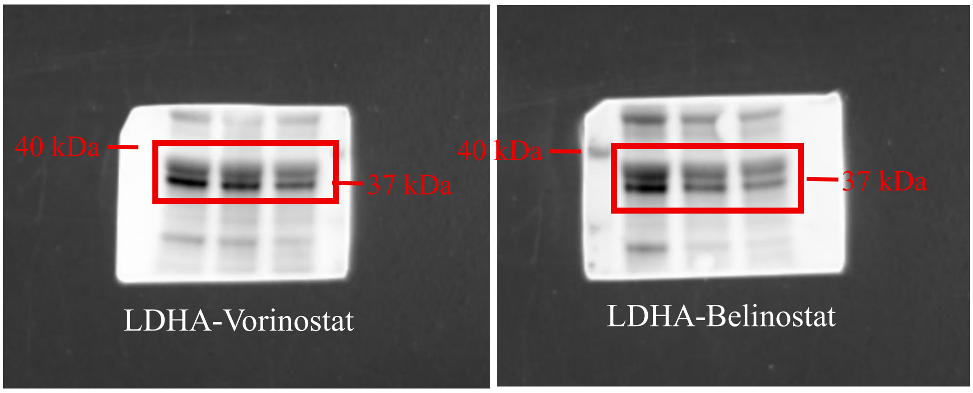

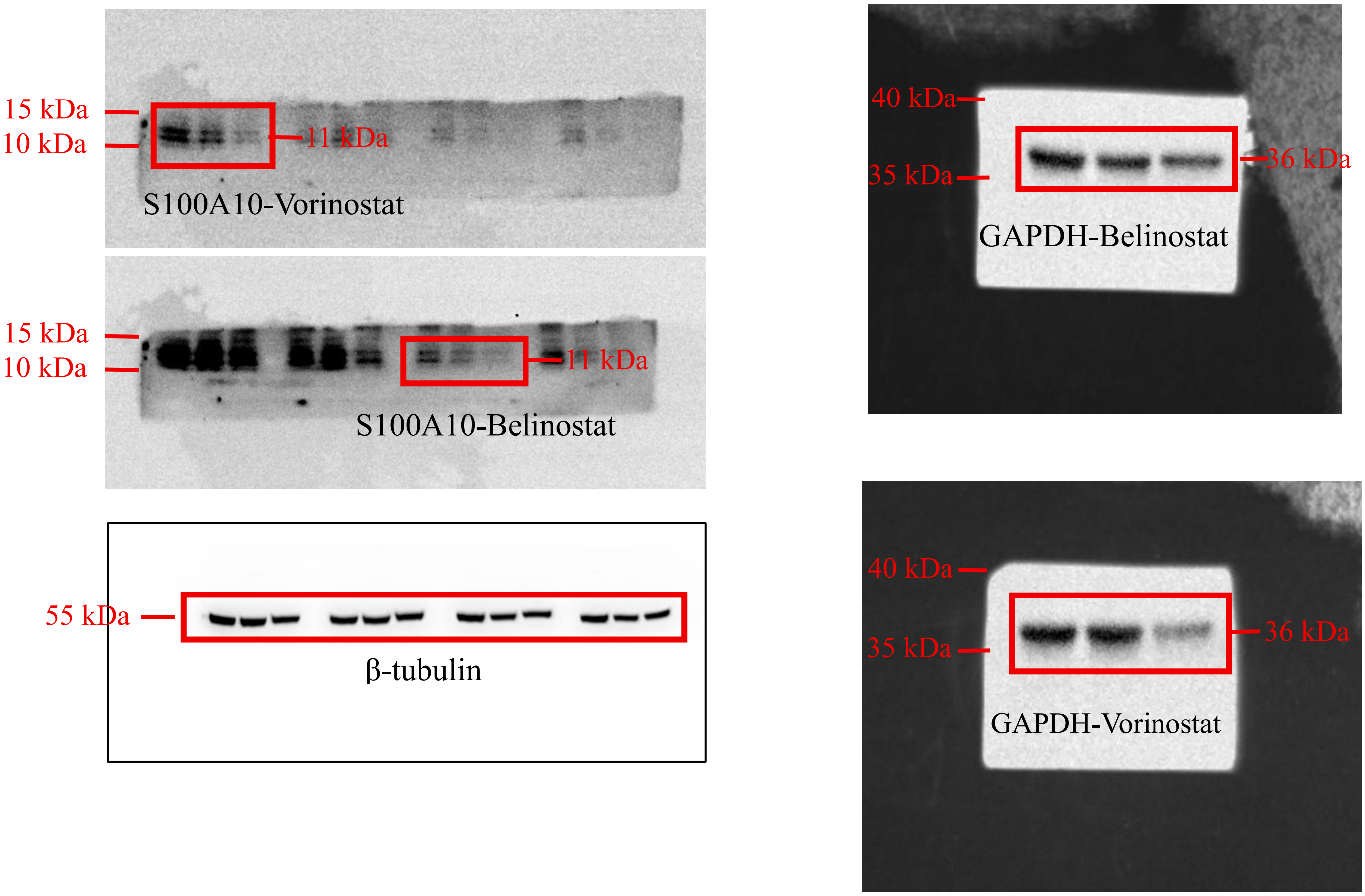
15 kDa
10 kDa
11 kDa
S100A10-Vorinostat
15 kDa
10 kDa
11 kDa
S100A10-Belinostat
55 kDa
β-tubulin
40 kDa
35 kDa
36 kDa
GAPDH-Belinostat
40 kDa
35 kDa
36 kDa
GAPDH-Vorinostat
