# Supplementary figures and images for: Molecular architecture of the tumor microenvironment caused by BRCA1 and BRCA2 somatic mutations in human lung adenocarcinoma
Source: eLife. 2026 May 26;15:RP110662. doi: 10.7554/eLife.110662 (PMC13211877; doi:10.7554/eLife.110662)

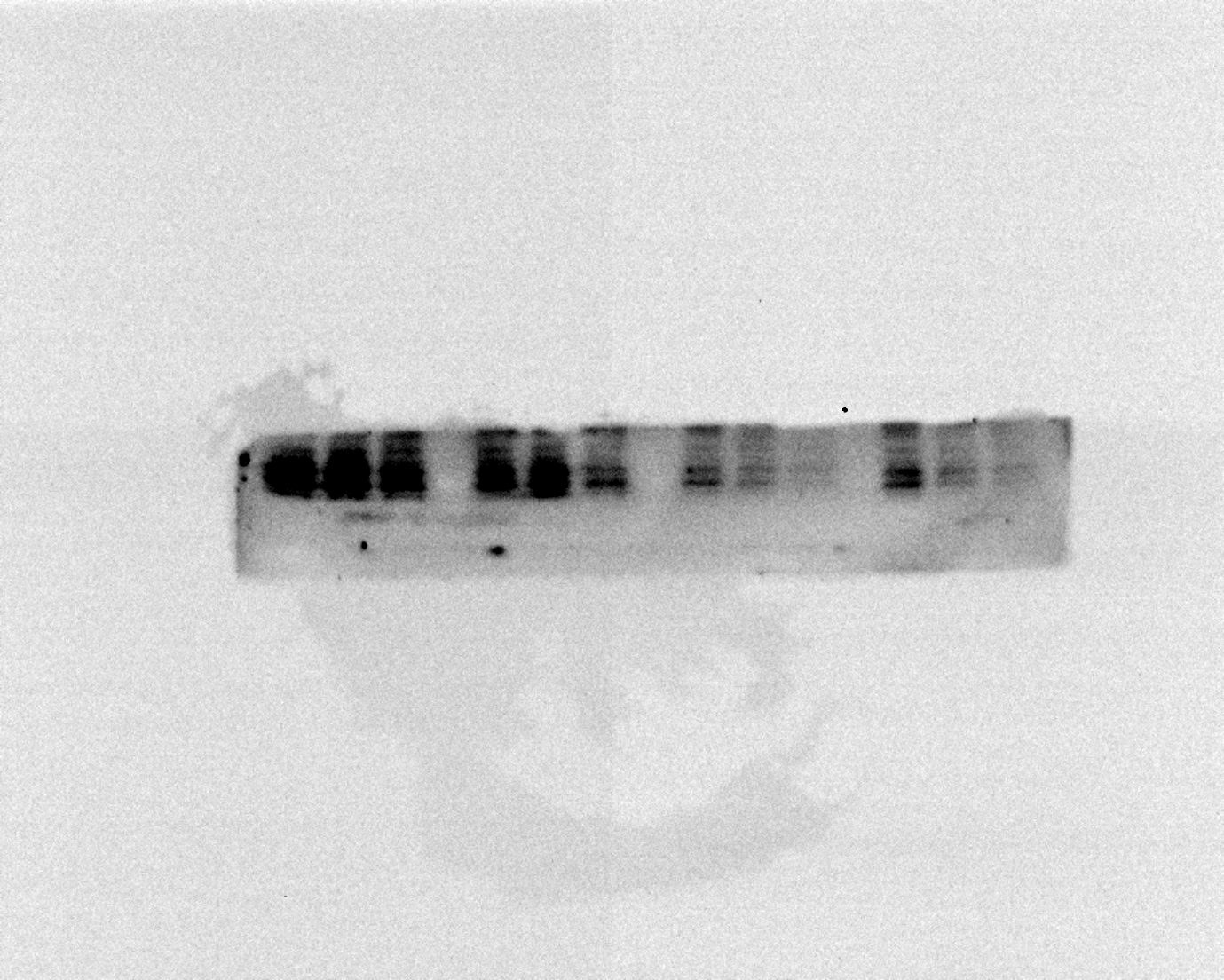

Supplement: Figure 7—source data 2. [file elife-110662-fig7-data2.zip › Figure 7-source data 2/Drug treatment 48h/S100A10-Belinostat.tif]

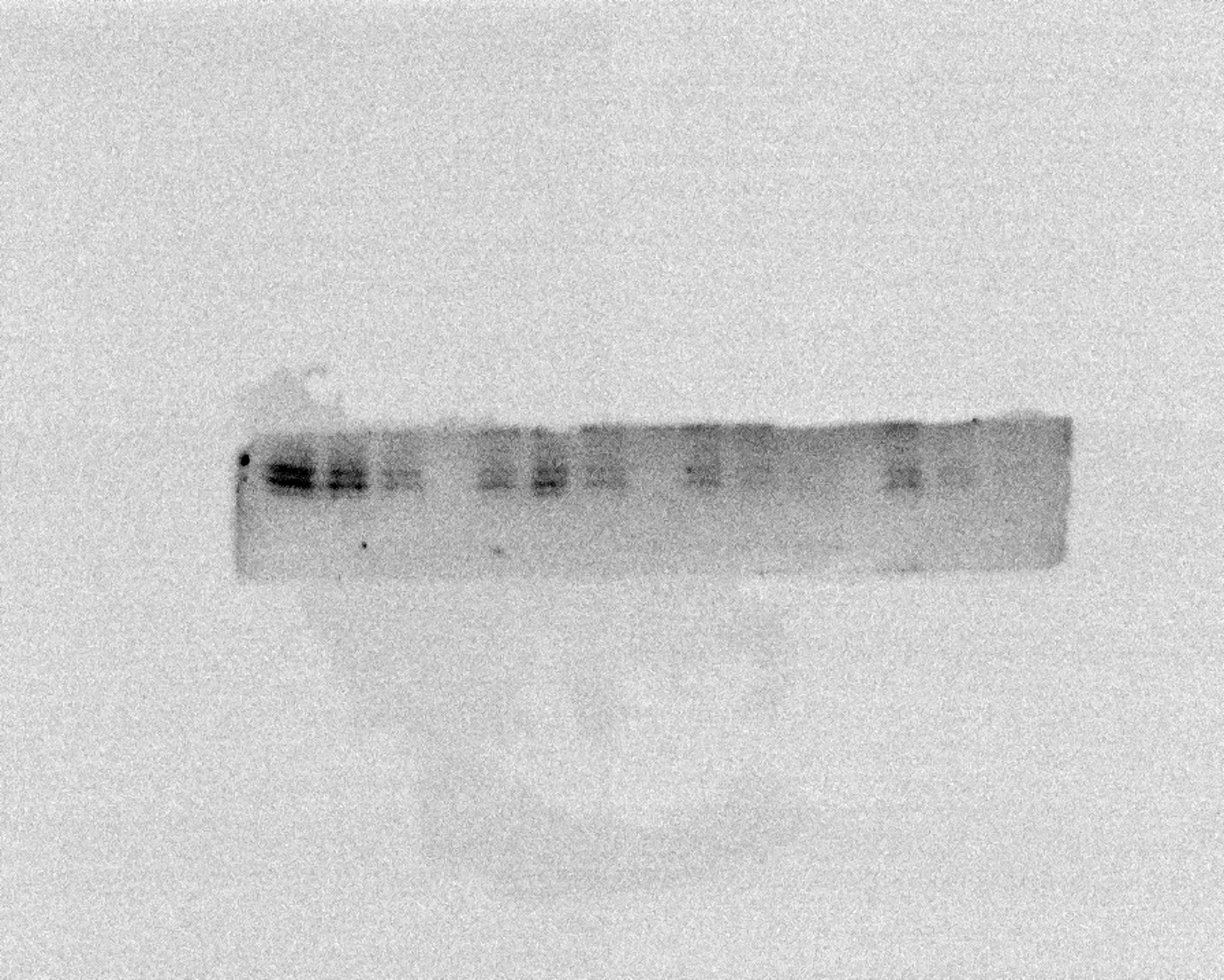

Supplement: Figure 7—source data 2. [file elife-110662-fig7-data2.zip › Figure 7-source data 2/Drug treatment 48h/S100A10-Vorinostat.tif]

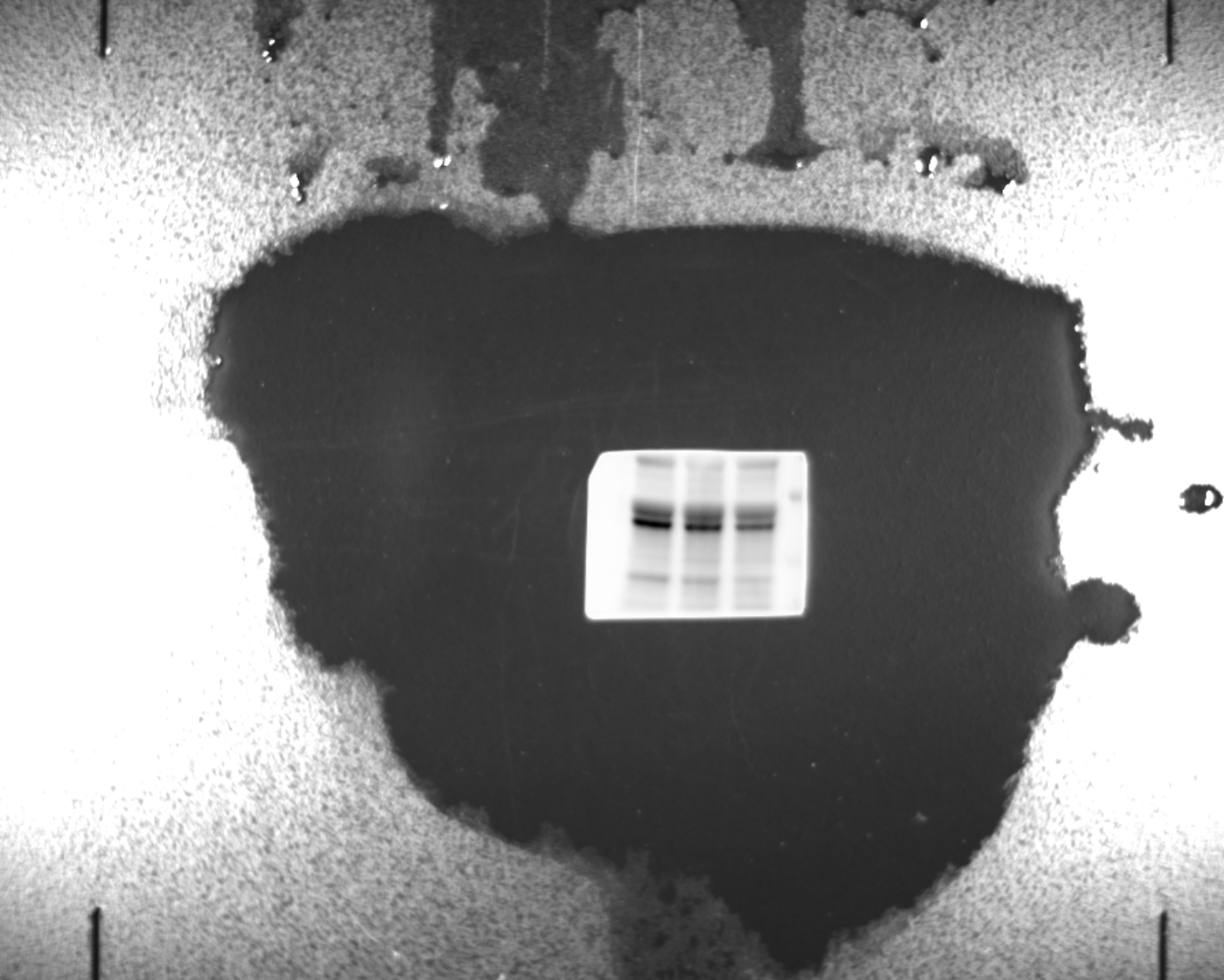

Supplement: Figure 7—source data 2. [file elife-110662-fig7-data2.zip › Figure 7-source data 2/Drug treatment 48h/LDHA-Vorilinostat.tif]

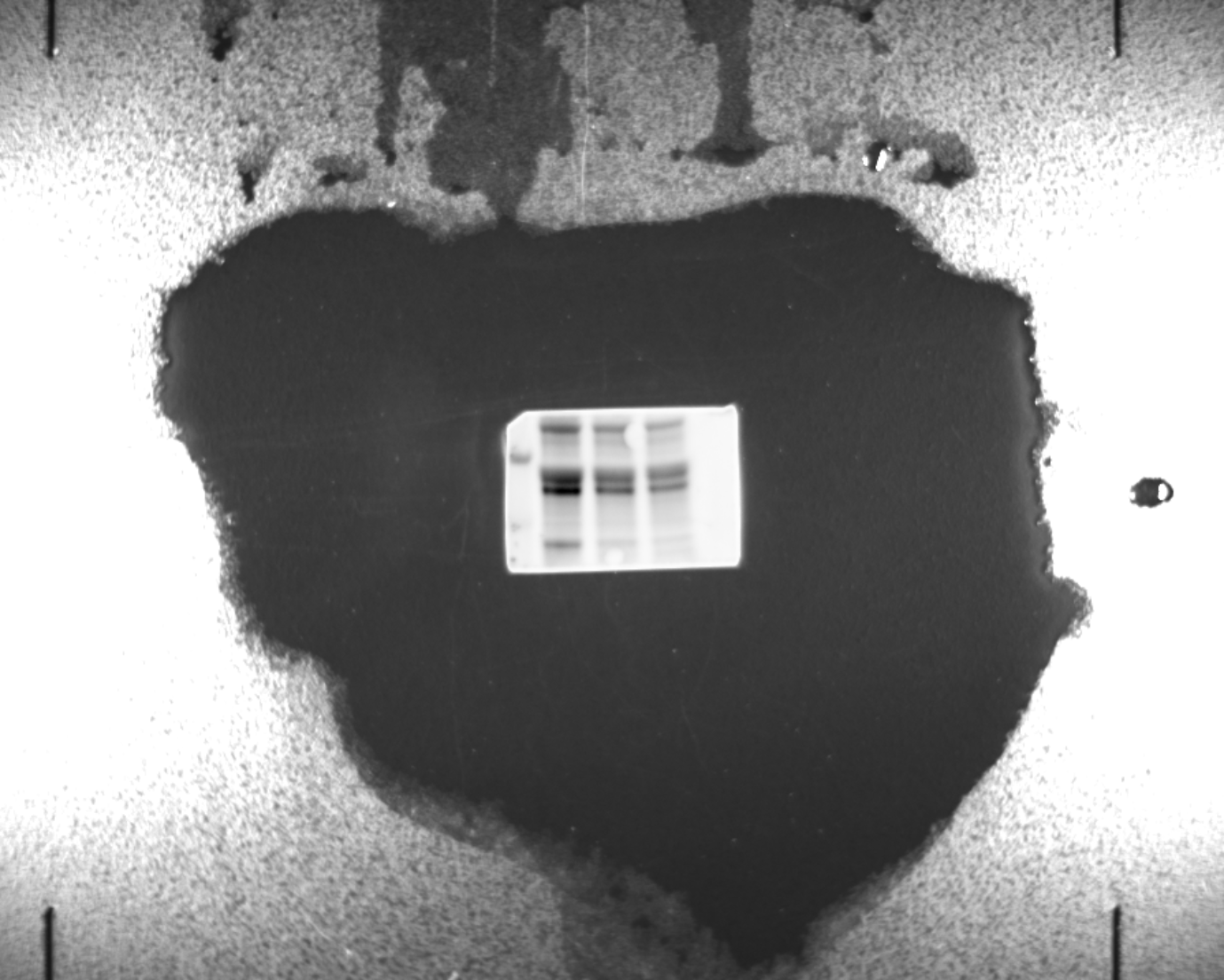

Supplement: Figure 7—source data 2. [file elife-110662-fig7-data2.zip › Figure 7-source data 2/Drug treatment 48h/LDHA-Belinostst.tif]

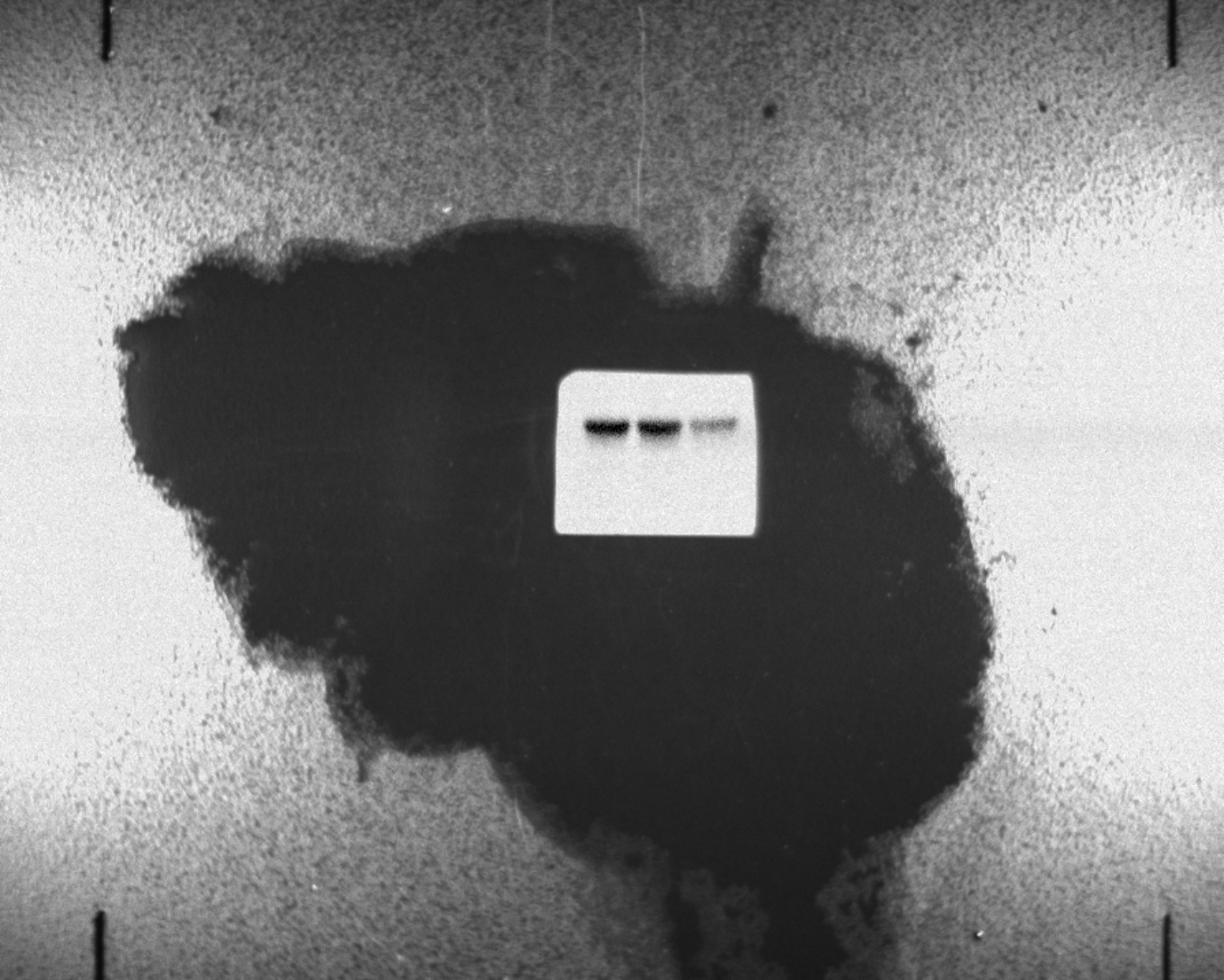

Supplement: Figure 7—source data 2. [file elife-110662-fig7-data2.zip › Figure 7-source data 2/Drug treatment 48h/GAPDH-Vorilinostat.tif]

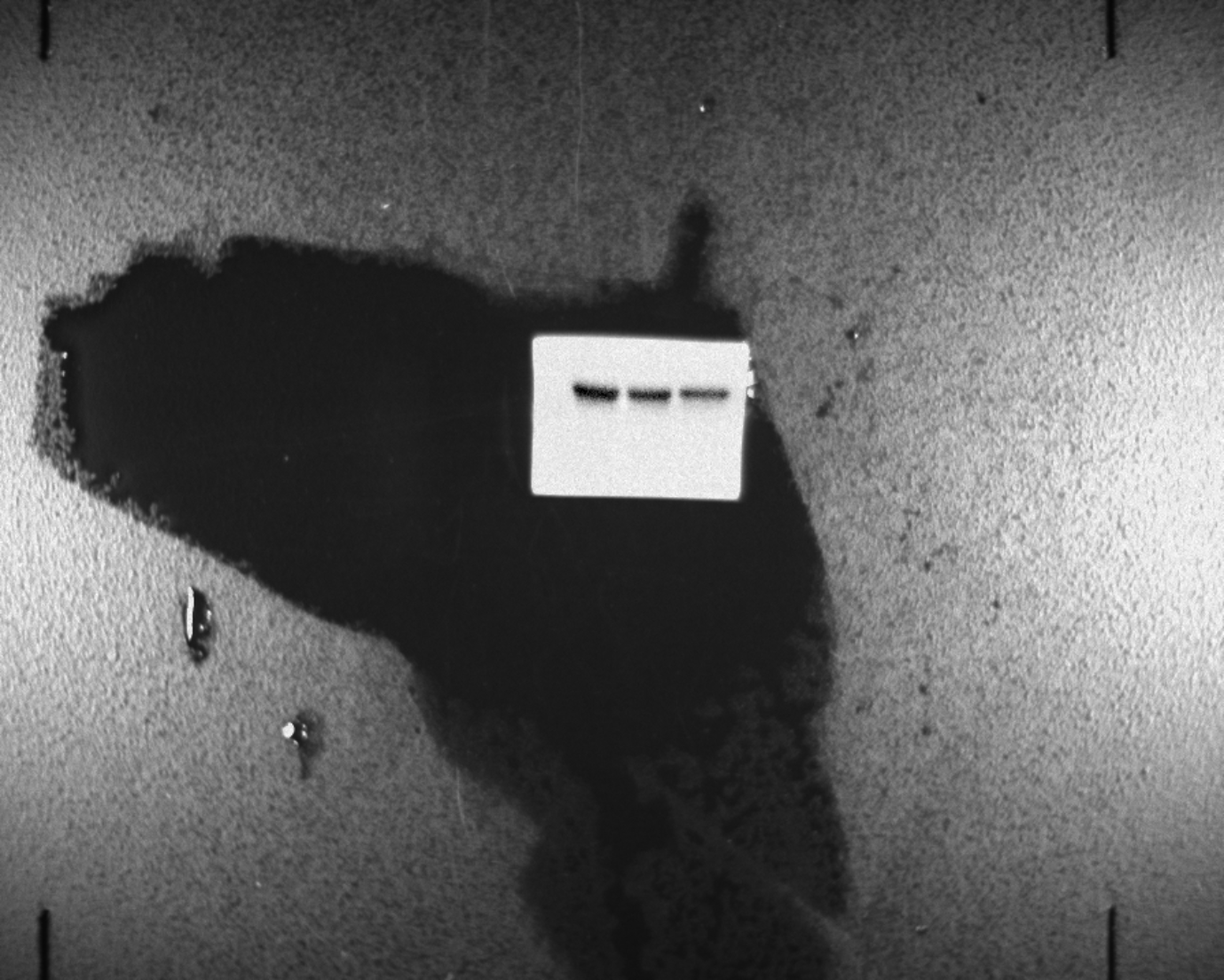

Supplement: Figure 7—source data 2. [file elife-110662-fig7-data2.zip › Figure 7-source data 2/Drug treatment 48h/GAPDH-Belinostat.tif]

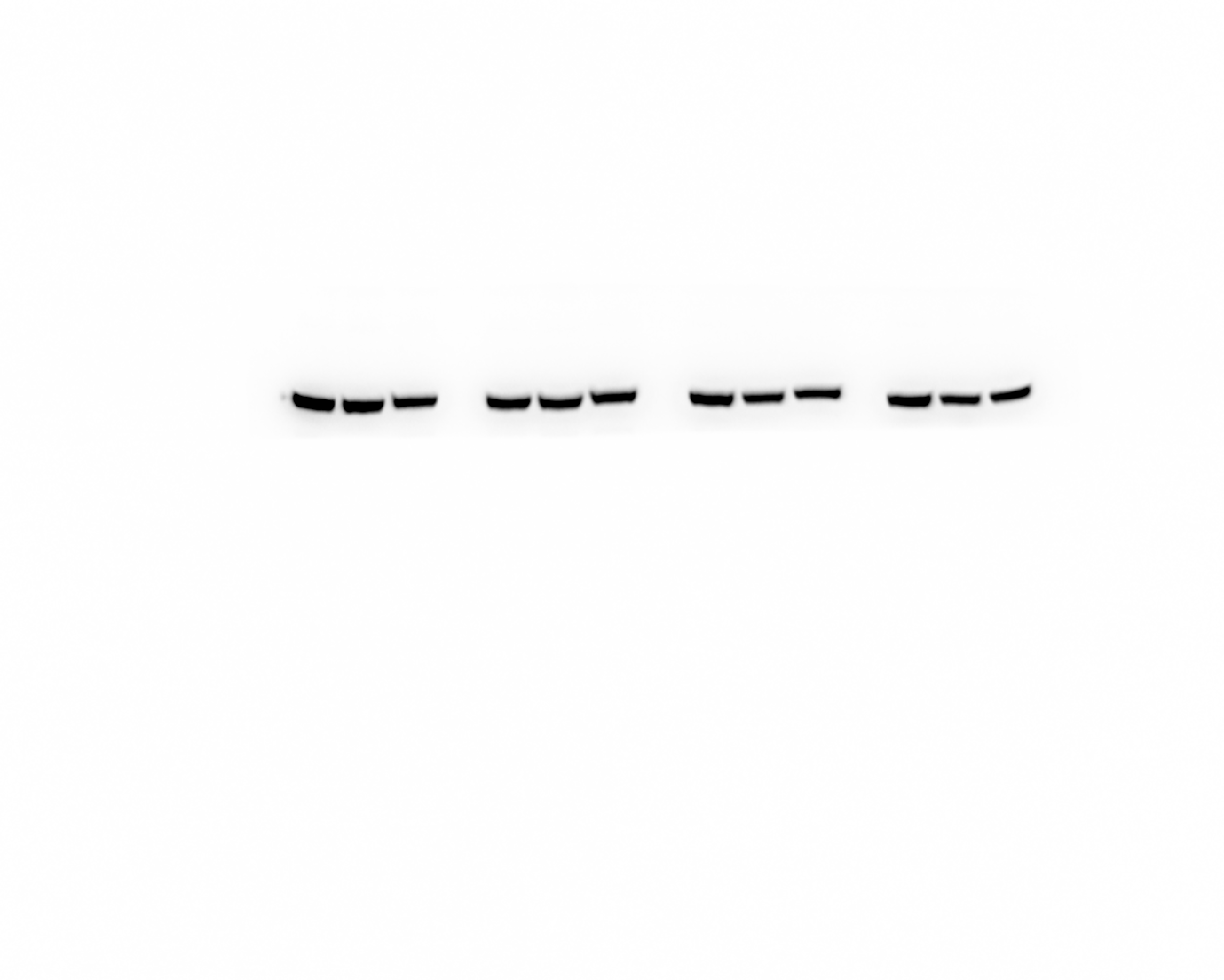

Supplement: Figure 7—source data 2. [file elife-110662-fig7-data2.zip › Figure 7-source data 2/Drug treatment 48h/B-tubulin.tif]

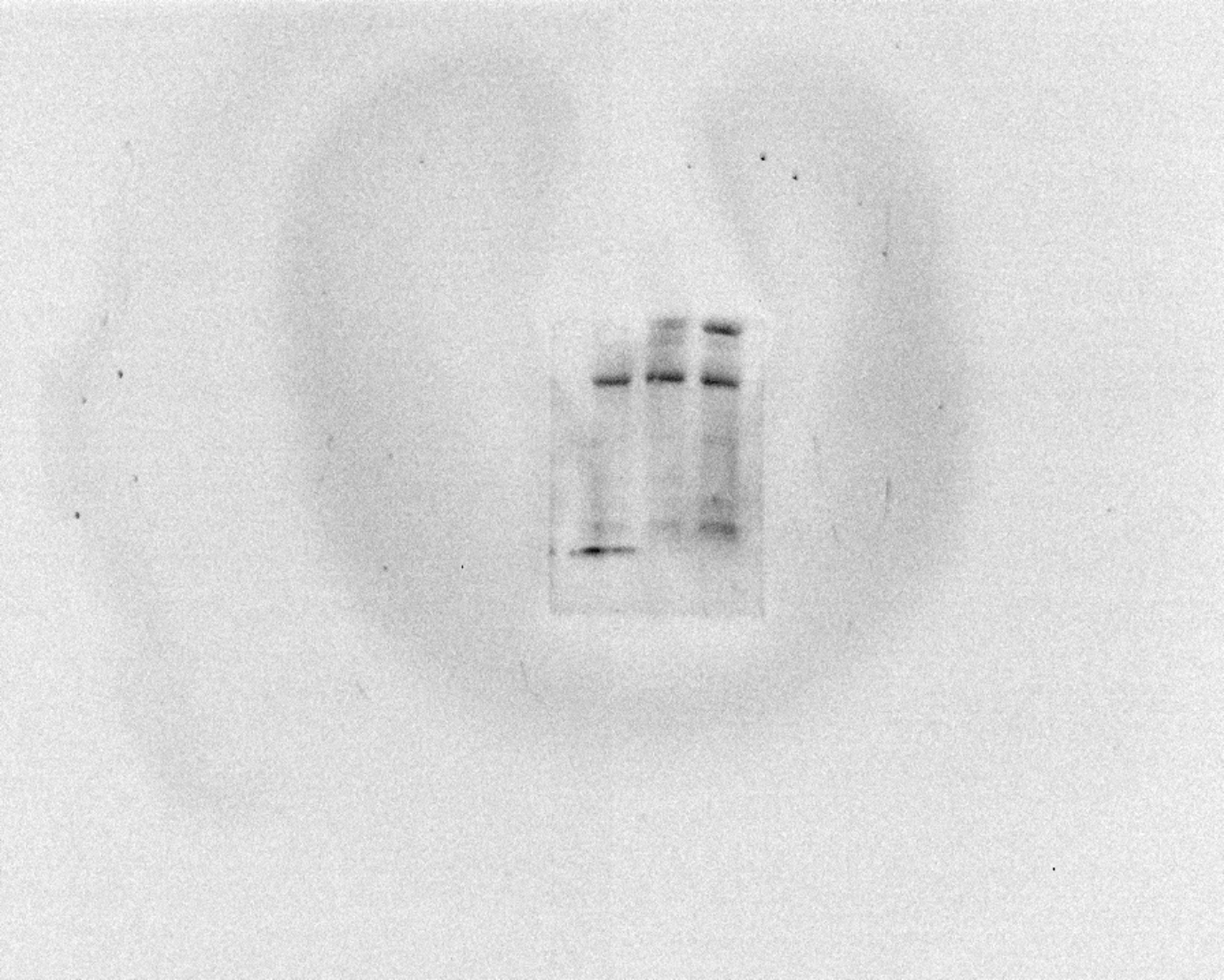

Supplement: Figure 7—source data 2. [file elife-110662-fig7-data2.zip › Figure 7-source data 2/shRNA/S100A10.tif]

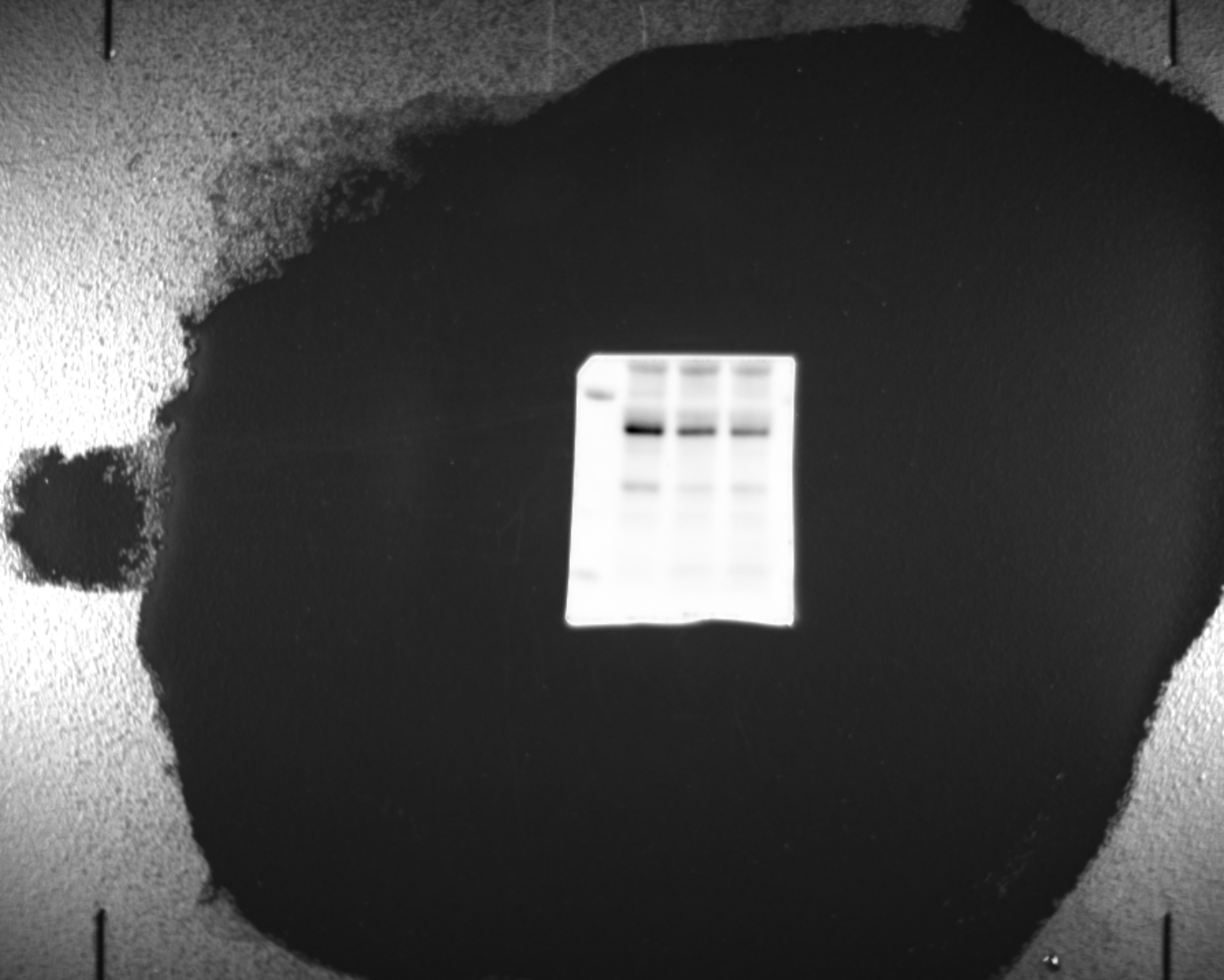

Supplement: Figure 7—source data 2. [file elife-110662-fig7-data2.zip › Figure 7-source data 2/shRNA/LDHA.tif]

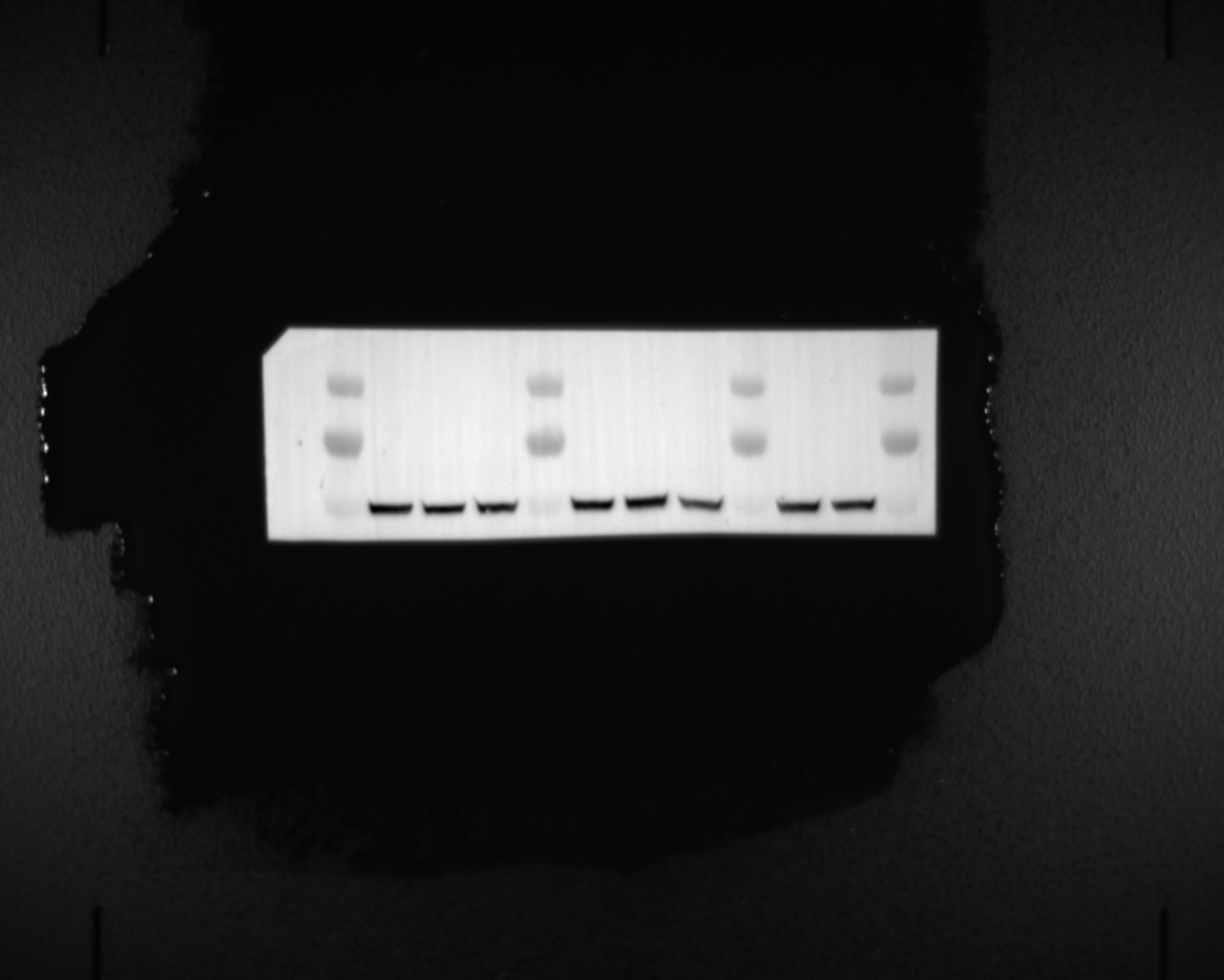

Supplement: Figure 7—source data 2. [file elife-110662-fig7-data2.zip › Figure 7-source data 2/shRNA/B-tubulin.tif]

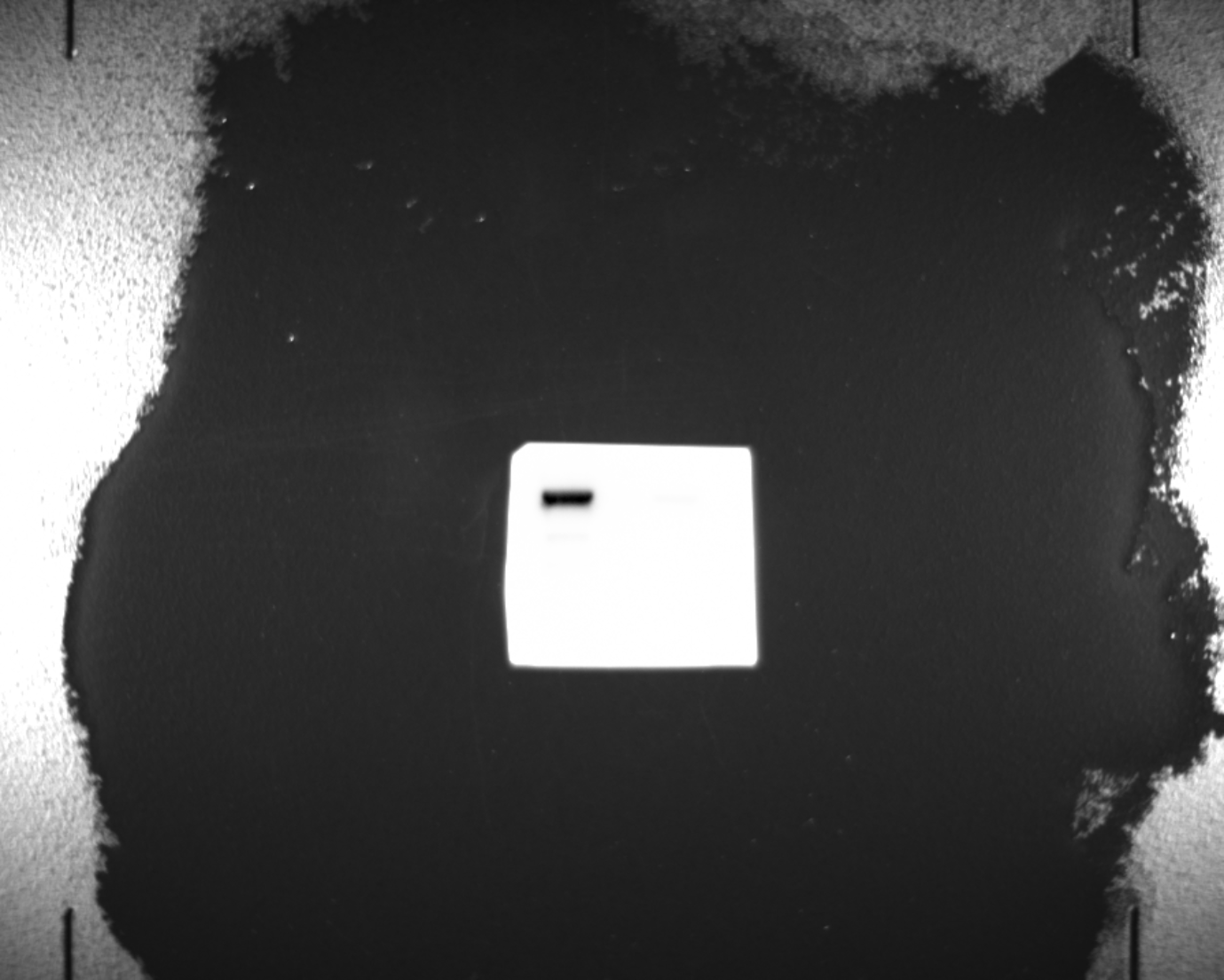

Supplement: Figure 7—source data 2. [file elife-110662-fig7-data2.zip › Figure 7-source data 2/shRNA/GAPDH.tif]
